# Connected Health in Hypertension Management

**DOI:** 10.3389/fcvm.2019.00076

**Published:** 2019-06-13

**Authors:** Stefano Omboni

**Affiliations:** ^1^Clinical Research Unit, Italian Institute of Telemedicine, Varese, Italy; ^2^Scientific Research Department of Cardiology, Science and Technology Park for Biomedicine, Sechenov First Moscow State Medical University, Moscow, Russia

**Keywords:** e-health, telehealth, telemedicine, telecare, telemonitoring, m-health, hypertension, blood pressure

## Abstract

e-health is defined as the use of communication and information technologies (ICT) to manage patients and their health in a more efficient way, with the aim of improving the overall quality of care. Healthcare services relying on telehealth (or telemedicine) and mobile health (m-health) are the most popular e-health tools used by healthcare professionals and consumers. These applications allow the exchange of medical data between patients and their doctors or among healthcare professionals, mainly through the Internet, and are used to provide healthcare services remotely (so-called “*connected health*”). The most popular telemedicine application in the field of hypertension is blood pressure telemonitoring (BPT), which enables transmission of BP and various clinical information from patients' homes or from the community to the doctor's surgery or the hospital. Numerous randomized controlled trials have documented a significant BP reduction combined with an intensification and optimization of the use of antihypertensive medications in patients making use of BPT plus remote counseling by a case manager, with the supervision of a doctor or a community pharmacist (telepharmacy). The major benefits of BPT are usually observed in high-risk patients. BPT can also be based on m-health wireless solutions, provided with educational support, medication trackers and reminders, and teleconsultation. In this context, BPT may favor patient's self-management, as an adjunct to the doctor's intervention, and foster patient's participation in medical decision making, with consequent improvement in BP control and increase in medication adherence. In conclusion, e-health solutions, and in particular telemedicine, are increasingly attaining a key position in the management of the hypertensive patient, with an enormous potential in terms of improvement of the quality of the delivered care, increase in the chance of a successful BP control and effective prevention of cardiovascular diseases.

## Introduction

Despite the progress in the effectiveness of screening, diagnosis, and treatment, elevated blood pressure (BP) continues to be the leading risk factor for death and disability in developed and developing countries (1). Hypertension currently affects about one billion people globally and it is predicted to be the major modifiable risk for non-communicable disease in the next two decades (1–3).

Although in the past few years a steady increase in the rates of high BP awareness and control has been observed, yet one-third of adults with hypertension are unaware of the condition, and among patients with prevalent hypertension, nearly half still have their BP uncontrolled (4–6). The significant increase in hypertension prevalence in recent years, particularly in the young (6), recommends that cardiovascular (CV) risk factors, including BP, are detected and dealt with more promptly in the population, particularly in those subjects with limited access to health care. Obtaining an accurate BP measurement is also critical for an effective estimation of patient's BP level and for tailoring the therapeutic intervention: in order to achieve this goal, doctors should rely more often on out-of-office BP measurements. Important hindrances to a proper detection and management of high BP are also the physician's inertia and the low patient's adherence to therapeutic plans.

In order to effectively tackle the hypertension burden the traditional healthcare model of hypertension management based on periodic office visits must be complemented by a modern approach which relies on new technologies and which contemplates patient's self-management as a mean to improve BP control. In this context, digital health or e-health represents an emerging field where medical informatics, public health, and business interact in a complex way in order to allow remote delivery of health services and health-related information and improve health care on a large scale.

In this review we will examine how e-health can help to improve hypertension management, focusing in particular on those tools used to provide healthcare services remotely (the so-called “*connected health*”). We will present the current evidence regarding the clinical efficacy of the most extensively tested connected health interventions in the field of hypertension management: blood pressure telemonitoring (BPT) and telepharmacy. We will also discuss how emerging mobile technologies may help to enhance connections between patients and healthcare providers and positively affect hypertension management.

## The Patient-Centered Model and the Personalization of Care

According to a modern model of hypertension management, the patient and not his/her disease has a central role and is directly involved in his/her health care management in collaboration with the physician, family, and community, each other interacting in different ways to influence and support health decision (7). This approach also emphasizes that patients with the same disease are nonetheless different from one another, due to differences in genetic predisposition as well as underlying mechanisms for high BP. Thus, different subjects may respond differently to the same antihypertensive treatment and a traditional population-based approach may not be effective. Rather an individualized or personalized approach is required, according to a modern medical model often referred to as “precision medicine” (8).

A key point for successful high BP management and “*whole-person*” comprehensive care according to a personalized patient-centered model of care is the use of health information technologies, which may help in creating networks involving various healthcare professionals (e.g., physician, nurse, pharmacist) and can make the communication among the care team and the patient more efficient, favorably impacting on patient's health, as we will further discuss in our review of the literature.

## E-health and Telehealth in Patient Management

### Types of e-health Services

e-health involves a broad group of activities that use communication and information technologies (ICT) to store, retrieve, share and exchange health-related information for prevention, diagnosis, treatment, monitoring, educational, and administrative purposes (9). The technological development of the last two decades has broadened the meaning of the term e-health, originally limited to activities carried out at the local site, and has expanded it also to the electronic or digital health processes involving the Internet and healthcare delivery over a distance (more appropriately defined as telehealth or telemedicine). The most typical services which can be provided with e-health and the corresponding digital models are summarized in Table 1.

**Table 1:** Main categories of e-health services and their practical applications.

**e-health categories**
EHRs Administrative and clinical information systems (PIS) Clinical decision support tools Web-based technologies and services Virtual healthcare (teleconsultation and remote diagnosis), robotics, computer-assisted surgery Medical research support technologies (grid technologies) Telehealth (telemedicine, telecare, m-health)
**e-health applications**
On-line medical records On-line appointment booking Social media and online forums: online discussion groups for patients and healthcare professionals (peer-to-peer support) e-learning: web-based interactive education programs on health-related topics for patients (e.g., cardiovascular risk factors, including high BP, lifestyle) and continuing medical education for healthcare professionals Medical imaging and diagnostic (e.g., vascular and cardiac ultrasonography) Interactive system check/message Wearable sensors (e.g., cuff-less BP monitors) Mobile health apps for patient's self-management, training, and education Digital clinics: with synchronous (live interaction video, telephone consultations, web chat, telemonitoring) or asynchronous communication (e-mail, SMS and text messages) On-line prescription and drug dispensation

*EHR, Electronic Health Record; PIS, Patient Information Systems; BP, Blood Pressure; SMS, Short Message Service*.

The most common e-health tool is the Electronic Health Records (EHRs), namely a software used to store a patient's clinical history (test results, medication, and general clinical history) and share it online with other providers outside the practice in order to enable the communication of patient data between different healthcare professionals and to support clinical actions. In some settings, like the hospital or primary care facilities, EHRs are integrated into Patient Information Systems (PIS) and are used to support both the administrative and clinical activities.

Recently, the growth of the Internet has fostered the diffusion of web-based e-health services such as interactive education and disease prevention programs, online discussion groups for patients.

Healthcare services can be provided to patients and other health professionals through virtual clinics, which enable teleconsultation and videoconferencing, exchange of diagnostic images, and in some cases computer-assisted surgery. Finally, e-health can also be employed as support to medical research, using grid technologies which allow powerful computing and data management capabilities to handle large amounts of heterogeneous data.

### Telehealth: Definition and Applications

The most popular e-health services among healthcare professionals and the consumers are those based on telehealth and mobile health, namely applications which are limited to healthcare over a distance.

Telehealth basically refers to the use of ICT to deliver healthcare and clinical services and medical education from one site to another, in order to provide more prompt and efficient diagnosis and clinical care (10). The term telemedicine is usually considered as a synonym of telehealth (and we will use the two terms interchangeably in our review, as suggested by the American Telemedicine Association) (11). When mobile communication devices (e.g., smartphones or tablets) are used to exchange data or information between doctors and patients, the term mobile health (m-health) is employed and this category considered a subset of telehealth (12). Typically, m-health applications are used to track and process a large volume of lifestyle and well-being information.

**Figure 1:**
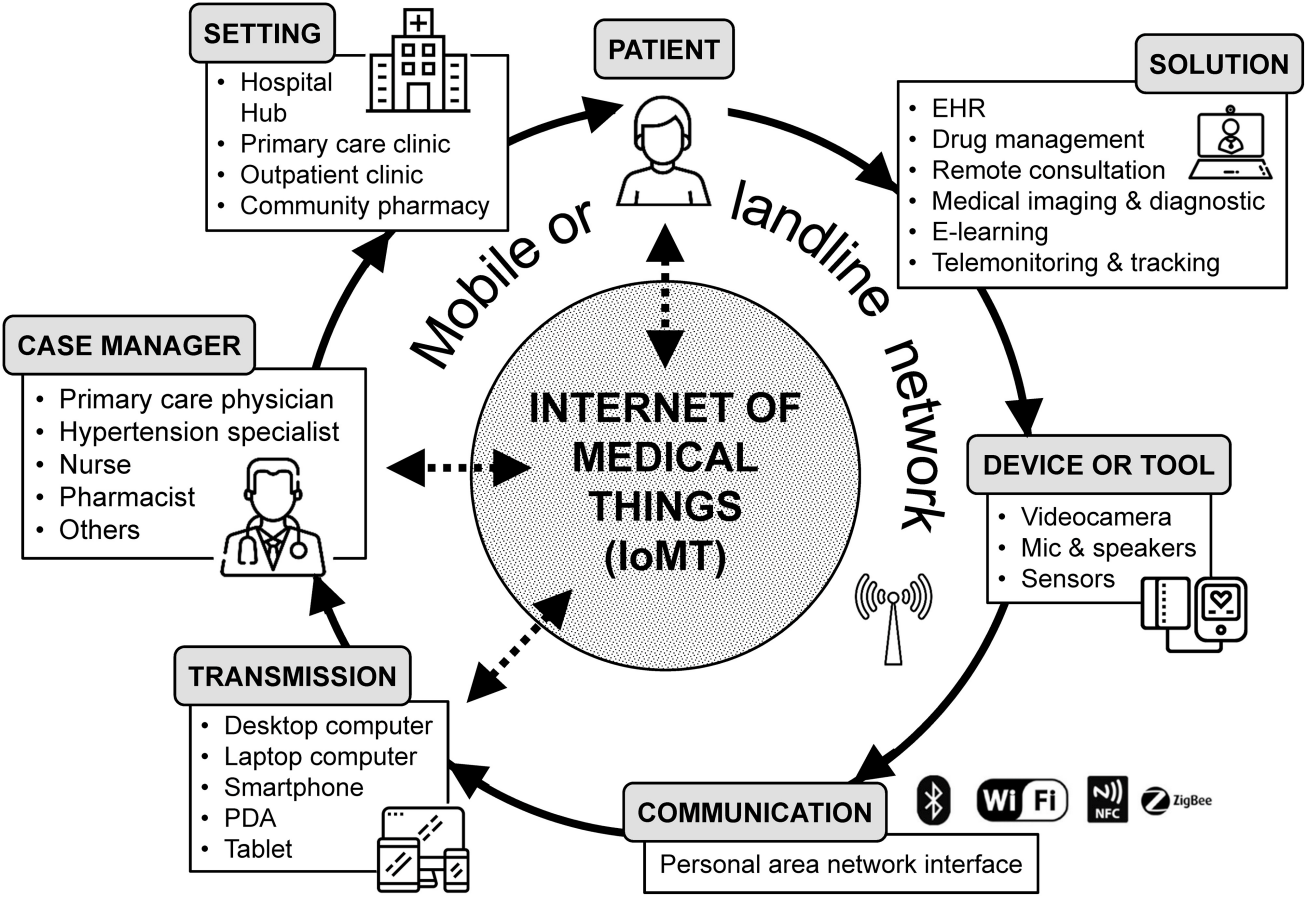
Diagram of most common telehealth services and their workflow. EHR, Electronic Health Record; NFC, Near Field Communication; PDA, Personal Digital Assistant; IoMT, Internet of Medical Things.

An exemplification diagram of telehealth services and their workflow is depicted in Figure 1. Currently, the most common delivery mechanism for telehealth is the connection between users and healthcare facilities through the web. The use of a web-based shared platform favors collaborative care and interactions between stakeholders, according to a “*closed-loop*” healthcare model. This is also called “*Internet-of-Medical-Things*” (IoMT), a connected infrastructure of health systems and services, which is devised to identify issues before they become critical and allow early intervention by caregivers (13). Dedicated high-speed links between sites (point-to-point connection) are alternatives to the more modern Internet-based approach to telehealth; however, these solutions are now less popular than in the past and used for particular applications such as the transmission of radiological images, stroke assessment, mental health and intensive care services.

### Diffusion of Telehealth Services Worldwide

Unfortunately, exact estimates of users and market size for telehealth vary widely. However, it is out of question that telehealth is a rapidly growing component of healthcare worldwide and, due to currently poor implementation, financial challenges and lack of physician support, it has yet to reach its tipping point. Around the world, 7 million patients are managed by telehealth solutions, but these numbers may be underestimated. The most popular telehealth applications are in the field of radiology and cardiology, and patients are predominantly diagnosed with cardiovascular diseases such as heart failure or hypertension, and diabetes (14).

North America currently dominates the market of telehealth, followed by Europe (15, 16). In the United States of America (USA) there are currently ~200 telemedicine networks with 3,500 service sites and over half of all US hospitals have adopted some form of connected health to monitor their patients (11). In Europe, 83% of countries have telemedicine services implemented, the main being teleconsultation (73%), followed by telediagnosis and telemonitoring (67%) (17). The most common areas where these services are offered in Europe are radiology (87%) and cardiology (80%). Diagnosis is the main purpose of referring to telehealth applications (67%), followed by emergency care and vital sign monitoring (53%).

The global telemedicine market was valued at 38 billion of USD in 2018, and it is estimated to be valued at 104 billion USD in 2024, witnessing a compound annual growth rate of 18% (15). According to a recent survey of the European market, EHR, picture archiving and communication systems (PACS), and telehealth services are gaining increasing importance among the e-health solutions (16). In particular telemonitoring appears to be the fastest growing application of telehealth and it is expected to help in augmenting the growth of the overall e-health market.

## Blood Pressure Telemonitoring

### Devices and Technologies

BPT is the most popular telehealth application for hypertension management. Basically, it allows remote data transmission of BP measurements and other information on patients' health status from their homes or from a professional healthcare setting (e.g., community pharmacy or primary care clinic) to the doctor's surgery or the hospital. Automated upper-arm BP monitors are usually employed to collect casual BP readings in the surgery, or multiple measurements over the 24-h, in ambulatory conditions, or during consecutive days, at home, by self-measurement (18). Presently, “*cuffless*” devices based on wrist tonometry or finger photoplethysmography are growing in popularity, as extensively discussed in another section of this paper.

Table 2 summarizes the type of devices and technologies for BPT currently available on the market. The systems differ for the types of devices used for patient's data collection, mode of data transmission and reporting, and for additional features such as time reminders for BP measurement and pill intake, and automatic data reporting. In BPT solutions, BP measurements are saved in the memory of the device and then sent, synchronously or asynchronously, to a remote computer host. Data transmission is usually fulfilled via fixed-line or mobile broadband networks, and through the web, using encryption transmission protocols, which ensure data integrity and security. When data are received at the central telemedicine server they are stored and analyzed, with automatic generation of reports which are reviewed by case managers, before they are submitted to the reporting physician. Once a medical report is produced, it is forwarded to the patient and his/her managing general practitioner through a website, via e-mail or dedicated smartphone apps. During all these processes the case manager may also interact with the patient in order to obtain feedbacks on his/her health status and tailor the drug treatment according to the indications of the managing physician (so-called co-intervention or additional support).

**Table 2:** Technologies for blood pressure telemonitoring.

**Types of monitoring devices**
Upper-arm electronic automated devices (wired or wireless) Devices for monitoring multiple parameters such as ECG, pulse oximetry, body temperature, body weight, blood glucose, medication intake (so-called “medical tricorders”) Smartphone applications either paired with an external wireless BP monitor or allowing manual data entry Smartphone serving as cuffless BP monitors Wearable monitors for long-term surveillance (e.g., wrist tonometers or finger plethysmography)
**Modality of data communication and transmission**
Dedicated wireless devices with built-in mobile phone-based transmission systems (e.g., home hubs or smart boxes) Handheld devices (smartphones or tablets) with wireless communication linked to private (home) or public (community) wi-fi access points or to the mobile public network (transmission by landline or mobile network) Desktop or laptop computers linked to the BP measuring devices via wired (USB cable) or wireless connection (transmission usually by landline network)
**Type of data transmitted**
Immediate (synchronous) or periodic (asynchronous) automatic forward of encrypted data strings with proprietary or standard formats Manual data input by text messaging (SMS, social media applications such as WhatsApp, Facebook messenger, etc.) E-mail messaging (manual data input or list of readings sent as an attachment) Websites with dedicated forms allowing manual data input or manual upload of files
**Type of reporting**
Automatic: short diagnostic sentences based on validated algorithms, provided directly to the users Manual: diagnostic sentence manually implemented by a physician Hybrid: automatic reports needing confirmation by a physician

*BP, Blood Pressure; ECG, Electrocardiogram; USB, Universal Serial Bus; SMS, Short Text Messaging*.

### Evidence of Clinical Effectiveness

BPT is usually well-received by hypertensive patients (19). In 13 studies, including 1,662 patients, the adherence to BPT programs was high on average (76.8%, range 48–90%). In 10 studies, including 1,120 patients, 87.1% of the participants (range 69% to 100%) regarded the BPT technique as useful to manage their condition.

Concerning the clinical effectiveness, several randomized controlled trials have documented that the regular and prolonged use of BPT with telecounseling and case management under the supervision of a healthcare professional is associated with a significant BP reduction compared to usual care, this being particularly evident for high-risk patients.

A meta-analysis of randomized controlled studies including 23 comparisons and 7,037 hypertensive patients showed that regular BPT at home with a follow-up of at least 6 months is associated with greater reductions in both office [average and 95% confidence interval: 4.7 (6.2, 3.2) mmHg for systolic blood pressure (SBP) and 2.5 (3.3, 1.6) mmHg for diastolic blood pressure (DBP); *p* <0.001 for both] and 24-h ambulatory BP [3.5 (5.3, 1.6 mmHg for SBP with *p* < 0.001 and 1.4 (2.9, 0.0) mmHg for DBP with *p* = 0.051] as compared to usual care (periodic BP measurements and visits at the doctor's office, with no remote BP monitoring) (20). Patients resorting to BPT also had a higher probability of achieving a good office BP control [relative risk and 95% confidence interval: 1.16 (1.04, 1.29), *p* = 0.007]. The improved BP control obtained with BPT was associated with an intensification in the use of antihypertensive medications [+0.40 (0.17, 0.62), *p* < 0.001], but a frequency of doctor's office visits similar to that of the usual care group. The BPT group had significantly (*p* < 0.0001) larger expenses than the control group [+662.92 (+540.81, +785.04) euros per patient]. However, the expenditure was similar for the two groups when only medical costs were considered, and those related to the technology were not accounted for [−12.4 (−930.52, +906.23) euros, *p* = 0.767]. Use of BPT helped to improve the physical component of quality of life, but not the mental one.

Recently, an updated meta-analysis including 46 randomized controlled trials and 13,875 cases, confirmed the superiority of BPT on usual care in terms of improvement of BP control (21). At variance from the previous meta-analysis the new publication also compared the efficacy of BPT alone (home BP measurement with simple data transmission with no interaction or with a low intensity additional support) vs. BPT plus additional support (including face-to-face counseling, telecounseling, education, behavioral management, medication management with decision support, adherence contracts), showing a trend to a larger effect of the more intensive intervention in terms of office BP reduction [2.4 (4.9, 0.0) mmHg for SBP with *p* = 0.05 and 1.1 (2.3, +0.1) mmHg for DBP with *p* = 0.07]. This meta-analysis showed low-strength evidence of the efficacy of BPT for surrogate outcomes and did not show any significant difference for BP changes between short-term (<12 months) and long-term studies (>12 months).

Even after taking into account the intrinsic limitations of the meta-analytic approach, the results of the systematic reviews presented above suggest that BPT may represent a useful tool to improve hypertension management. Unfortunately, most of the current evidence comes from studies with a relatively short duration (<12 months). In the few studies looking at longer-term outcomes, no evidence of better or sustained effect could be provided. Additionally, the studies performed so far did not help to define the best BPT-based healthcare delivery model, because of the heterogeneity of interventions, technologies and study designs. Hence, properly designed, large-scale, prospective, controlled studies based on a validated technology are needed to better understand the long-term effects and sustainability of BPT. Such studies should be focused in particular on patients for which an optimal BP control is particularly difficult to attain, such as those at higher risk of developing a cardiovascular disorder. They should also investigate the potential additional benefits of BPT beyond BP control and its cost-effectiveness, which may support future reimbursement models.

### Home Blood Pressure Telemonitoring or Home Self-Blood Pressure Monitoring?

Data from population-based surveys show that approximately half of the hypertensive adults regularly engage in home self-BP monitoring (22, 23). Conclusive evidence exists that home BP monitoring is useful for the initial diagnosis and the long-term follow-up of treated hypertensive patients, with a positive impact on BP control and compliance to treatment and a larger predictive power than office BP (24, 25). Compared with conventional home self-BP monitoring, home BPT may improve the quality of data reporting and make interpretation by doctors easier (19). However, questions which remain still open are whether long-term home BPT may benefit BP control to a greater extent than conventional home self-BP monitoring and which additional intervention and level of intensity of the intervention beyond self-BP are most suitable to achieve an effective BP control. Since BPT is a complex and still expensive procedure, one may question that its benefit is only modest compared to home self-BP with no telemonitoring.

**Figure 2:**
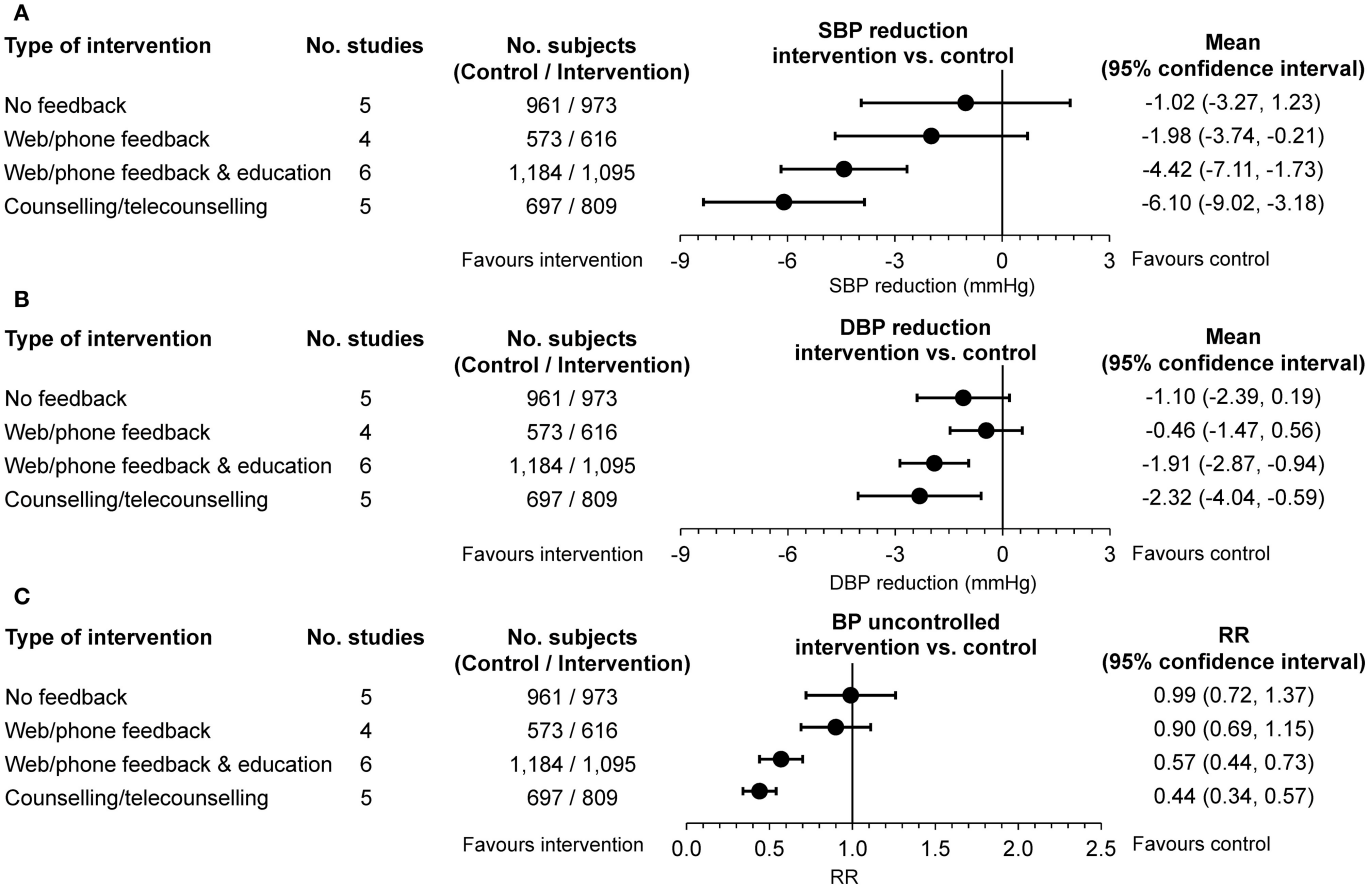
**(A)** Changes in office systolic blood pressure (SBP) and **(B)** diastolic blood pressure (DBP) and **(C)** rates of poor office blood pressure (BP) control at 12 months (intervention vs. usual care serving as control). Data are adjusted for age, sex, baseline clinic BP, and history of diabetes. Differences are shown as mean or relative risk (RR) and 95% confidence interval according to four levels of intervention (self-monitoring of BP with no feedback, self-monitoring with web or phone feedback, self-monitoring with web, or phone feedback and education, self-monitoring with counseling or telecounseling) [from (26) by permission].

An attempt to clarify this issue and bridge the knowledge gap has been provided by a recently published individual patient data meta-analysis of 15 randomized controlled studies based on self-monitoring of BP with an increasing intensity of intervention (self-monitoring with minimal additional contact, self-monitoring with automated feedback or support, self-monitoring with an active intervention or self-monitoring with significant tailored support) (26). As shown in Figure 2, self-monitoring was associated with reduced office BP compared to usual care at 12 months. However, this effect was strongly influenced by the intensity of the co-intervention, ranging from no effect with self-monitoring alone to the largest reduction when monitoring was combined with intensive support, including BPT. In this analysis, an attenuation of the effect of self-monitoring was observed in the few studies that followed patients for longer than 1 year, but data were sparse and heterogeneity significant (different inclusion criteria, self-monitoring regimes, and target BPs in included studies) to draw any definite conclusion. As a matter of fact, at 18 months the net effect of self-monitoring with counseling or telecounseling on SBP/DBP reduction was only marginally larger than that of self-monitoring with no feedback [−1.49 (−4.21, 1.23)/−1.11 (−2.49, 0.27) mmHg vs. −4.46 (−6.52, −2.40)/−1.65 (-3.63, 0.32) mmHg].

The results of this robust individual patient meta-analysis support and extend those of previous systematic reviews of 52 prospective comparative studies of home self-BP monitoring with or without additional support vs. usual care (27). Home self-BP monitoring alone was associated with statistically significant SBP/DBP reductions at 6 months (3.9/2.4 mmHg, 10 studies) but not at 12 months (1.5/0.8 mmHg, 8 studies). High-strength evidence supported a lower BP in case of BP monitoring plus additional support (including BPT), with reductions ranging from 3.4 to 8.9 mmHg for SBP and from 1.9 to 4.4 mmHg for DBP, at 12 months in 5 good-quality studies. In total, 13 trials directly compared the effectiveness of home self-BP monitoring with vs. without additional support (or with less intensive additional support). The authors of this systematic review concluded that the evidence was not strong enough to support a difference between the two approaches in terms of office BP lowering.

Thus, the results of the two largest meta-analyses available so far seem to suggest a possible additional effect of the BPT approach beyond self BP monitoring, with significantly larger and persistent (up to 12 months) BP reductions. However, more homogeneous and long-lasting interventions should be tested for effectiveness, also to provide information on the sustainability of the approach based on BPT.

### Potential Benefits of Blood Pressure Telemonitoring

All the BPT-based studies suggest that BPT may enhance hypertension management, improve patient outcomes, and reduce healthcare costs, particularly when long term follow-up are considered (28).

According to the evidence collected so-far, BPT has the potential for improving the effectiveness of BP control. As a matter of fact, in particular when based on home BP self-measurements, BPT shares the same advantage of out-of-office BP monitoring: the lack of alarm reaction during the measurement and the potential to obtain several reproducible BP measurements over the 24 h or during several days under daily life conditions (at home). Moreover, BPT allows the assessment of data in real time and the accelerated delivery of best practice when combined with decision-making strategies.

## Telepharmacy

### The Physician-Pharmacist Telehealth-Based Collaboration

Community pharmacists play a crucial role in hypertension management. In randomized controlled studies clinical pharmacy services offered to hypertensive patients have been found to enhance BP control and improve adherence to antihypertensive therapy as compared with usual care (29). In most of the studies performed so far in the field of hypertension, the interventions were provided by the pharmacists alone, whereas recent research supports the evidence that the multidisciplinary approach based on a physician-pharmacist collaborative practice and on a patient-centered model of care may be beneficial for appropriateness of medications use or for promotion of health and wellness of patients (30). In this context, a model of care based on telehealth (the so-called telepharmacy) may have the potential to expand the reach of the pharmacist's intervention and provide pharmacy operations and patient care at a distance with further benefits for hypertensive patients and their managing physicians (31). A variety of telepharmacy services for hypertensive patients are currently available, including phone contacts, medication dispensing, educational support, digital pill counts to track adherence, and telemonitoring, as summarized in Table 3.

**Table 3:** Most common currently available telepharmacy services for hypertension management.

Pharmacist-led telephonic clinics Medication counseling Drug review/monitoring (including adverse events and adherence) Provision of drug information Remote medication dispensing Medication therapy management Patient assessment and counseling (including teleconsultation) Virtual management within a multidisciplinary team Telemonitoring of BP and lab values (e.g., blood lipids, blood glucose) Automated text message reminders or phone calls Instructional and educational videos Educational websites

*BP, Blood Pressure [from (32) by permission]*.

Telepharmacy has the major advantage of making available in the community setting services for the hypertensive patients which are otherwise lacking, as in the case of rural or underserved areas, and of enhancing accessibility to healthcare providers and disease management by patients, ensuring continuity of care from the hospital to the community, with a constant support to primary care doctors. Among the several benefits of telepharmacy stands the fact the pharmacy is open 6–7 days a week, is usually at a walking distance from home or workplace, does not require an appointment to perform a test and the service is delivered promptly, in a professional context and at a lower cost than a medical facility.

### Evidence of Effectiveness of Telepharmacy for Hypertension Management

Studies on the efficacy of telepharmacy services for hypertension management have been mainly focused on home BPT, with a pharmacist-led intervention under the supervision of a physician (32).

However, there is some evidence on the impact of the pharmacist intervention on adherence and BP control through telephone services from two prospective controlled studies. In one study, 87 patients with both diabetes and hypertension received an intervention, based on a single initial phone call and 5 monthly follow-up calls (33). Those patients responding to more calls had significantly better adherence and less discontinuation during the 6 months following initial calls compared with controls who did not receive calls. Another study evaluated the effect of 3 months of counseling about medication and lifestyle issues by a pharmacist and health coach over the phone in 156 patients with uncontrolled hypertension (34). Patients were asked to complete an online questionnaire and to submit at least one blood pressure reading per week. At the end of the study, more patients in the intervention than in the control group had achieved target BP (71 vs. 31%, *p* < 0.001). Mean patient activation increased from 41.9 to 44.1 (*p* = 0.008), and the percentage of patients with low patient activation decreased from 15 to 6% (*p* = 0.003) in the digital-medicine group, suggesting that the intervention may empower and engage the patient in the management of his/her condition.

The strongest evidence of the clinical efficacy of telepharmacy services for the management of hypertension is limited to physician-pharmacist collaborative interventions based on home BPT plus patient education on lifestyle, drug therapy, and cardiovascular risk factors control (35–44).

**Figure 3:**
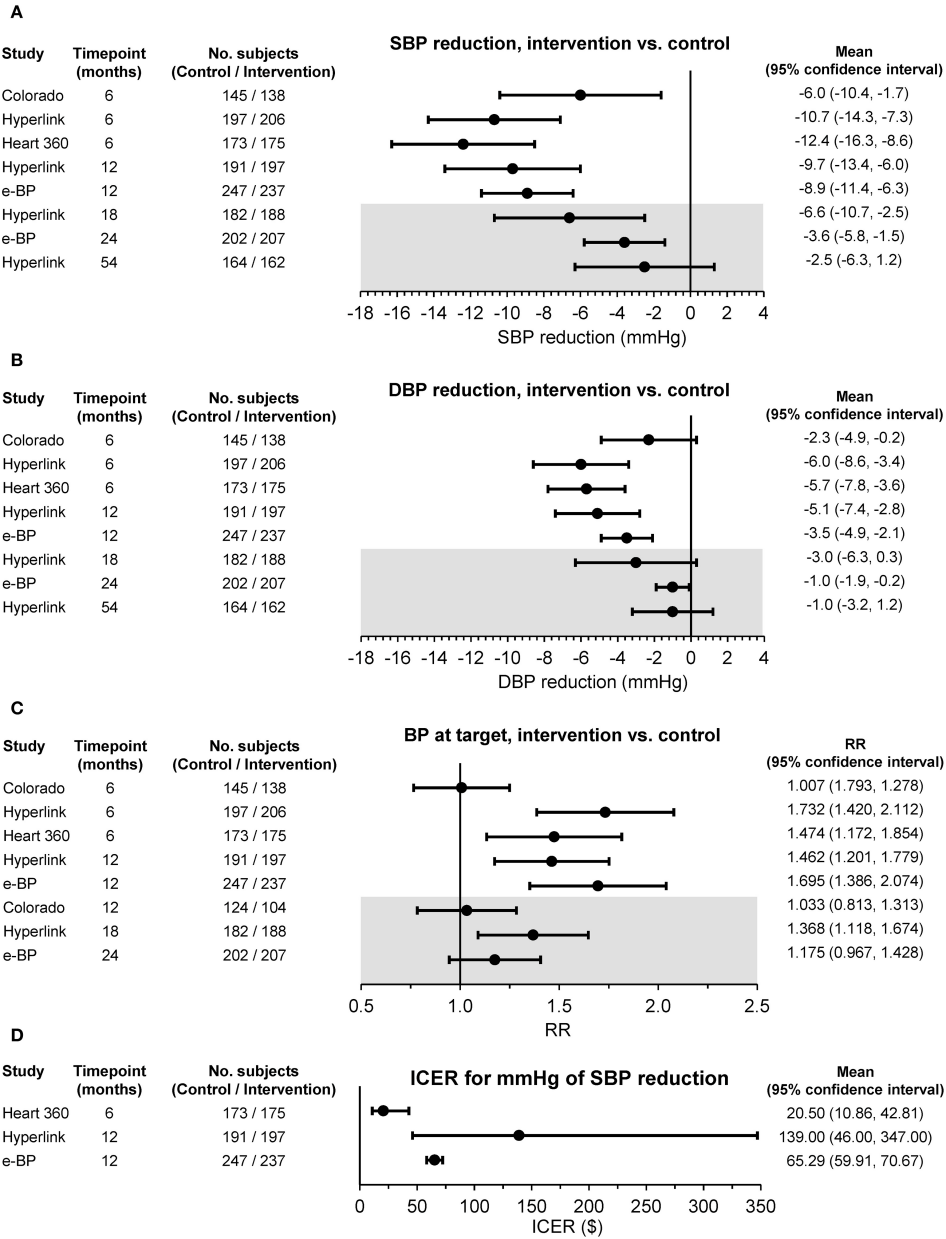
**(A)** Changes in office systolic blood pressure (SBP) and **(B)** diastolic blood pressure (DBP), **(C)** percentages of subjects with office blood pressure (BP) at target, and **(D)** incremental cost-effectiveness ratio (ICER) between the intervention and usual care group in randomized controlled studies based on pharmacist-led telehealth care. Differences are shown as mean or relative risk (RR) and 95% confidence interval at different time points. The gray insert refers to the period of the study following the withdrawal of the intervention [from (32) by permission].

The results of these trials documented a benefit of telehealth mainly in terms of improvement of BP control consequent to antihypertensive medication intensification and optimization. As shown in Figure 3, BP reductions and proportions of patients at target following a 6 to 12-month pharmacist's intervention were significantly larger than those observed in patients randomized to usual care. The benefit of the pharmacist's intervention was consistently reduced or even abolished months after its withdrawal, highlighting the importance of the sustainability of the intervention on the long-term. Interestingly, few of the studies also evaluated the cost-effectiveness of the intervention documenting that the improved BP control could be achieved at a relatively low cost compared with the usual care approach (Figure 3).

## m-health

### m-health Diffusion

There are currently 5.1 billion people around the world subscribed to mobile services, accounting for 67% of the global population (45). The number of smartphones is expected to steadily increase in the coming years and it has been projected that by 2025 there will be 5.8 billion smartphone users (71% of the population). The number of mobile Internet users is also growing, bringing the total number of subscribers from 3.6 billion in 2018 to 5.0 billion in 2025, closing the connectivity gap (45). Consequently, the number of Internet of Things (IoT) connections will triple, escalating from 9.1 to 25 billion (45).

This rapid increase of smartphone users and total connections is mirrored by an increase in the number of apps offering health services and information, and in the number of m-health apps' users. According to recent statistics on the app economy, there are currently 325 thousand health apps (health and fitness and medical apps) available on all major app stores (46). The app growth is fueled mostly by an increase of Android health apps, which have seen a growth rate of 50% from 2016 to 2017, compared to iOS health apps that have increased by 20%. In 2017 a total of 3.7 billion app downloads have been estimated. This trend indicates a growing demand for connectivity and health data exchange by consumers.

Some 50% of smartphone users gather health information on their mobile phones or tablets and 30% have at least one health app on their phone (47, 48). Diabetes and obesity continue to offer the best market potential for m-health solutions in the near future, but not the biggest markets. As a matter of fact, it is projected that diabetes and obesity will cover 70% and 35% of their market, with 422 and 600 million users, respectively, while apps related to cardiovascular diseases and hypertension will cover 15% and 25% of the markets, with 1 and 1.1 billion users, respectively (46). The most popular use case for health apps is the connection to doctors (30%), followed by diabetes management (27%), heart and vascular disease management (24%), and medication management (24%) (46).

### m-health Apps and Tools

Mobile-based applications can provide different functionalities to their users and today they are more than simple apps. Rather they represent the focal point and user interface within an ecosystem of connected devices and services, the so-called IoMT. In general, m-health encompasses a pool of solutions, which are used by consumers or healthcare providers, in order to monitor the patient's health status or to improve the patient's health outcomes. The most common functions provided by m-health apps and connected tools are listed in Table 4.

**Table 4:** Common functionalities provided by m-apps and modalities used to connect health digital data through m-apps.

**Functionalities**
Self-monitoring: tracking and recording of vital signs (not only BP but also body weight, lipid profile, blood glucose, physical activity, etc.) and drug intake Automatic feedback directed to the patient for positive reinforcement (e.g., in the form of illustrating BP trends) Reminders and alerts: mainly used to improve adherence to treatment and achieve other goals Educational information: about diseases and measuring procedures Logbooks of various activities Communication with health care providers
**Connected tools**
Wearables and tracking sensors: wearable BP and ECG monitors, fitness trackers, wristbands, smart watches, VR headsets Medical devices: non-wearable monitors (e.g., BP and blood glucose monitors, ECG devices, stethoscopes, spirometers, etc.) EHR: personal health and fitness data which can be exchanged between patients and healthcare professionals, hospitals, health insurances, etc. Health data aggregator: tools to allow access to structured health data and exchange between different platforms or apps and third-party health data (e.g., API service such as Apple Healthkit or Google Fit) Other tools: social networks, cloud data storage, data profiling, big data analytics

*BP, Blood Pressure; ECG, Electrocardiogram; VR, Virtual Reality; EHR, Electronic Health Record; API, Application Programming Interface*.

Apps for self-monitoring, tracking, and recording of vital signs, including for instance BP, body weight, lipid profile, blood glucose, physical activity, and drug intake, also in connection with medical devices or wearable sensors are quite popular applications of m-health. It has been estimated that currently, 42% of m-health apps connect to sensors and wearables and it has been estimated that in the next 5 years sensors built into devices (e.g., accelerometers, and heart rate or BP measured with phone's camera), rather than wristbands or smart watches, will be the most relevant category of tools connected to m-apps (49).

m-health apps can provide automatic patient-directed feedbacks, for instance in the form of illustrating patient's trend (BP in the case of the hypertensive patient) or overcoming of preset thresholds, and have the function of positive reinforcement of doctor's prescription and recommendation. Reminders and alerts can be forwarded through patients with the main purpose of improving adherence to treatment or compliance with the measurement of some parameters, such as BP. m-health apps can be used to provide educational information about the disease and measuring procedures to patients and healthcare professionals and to record the health status and the progress achieved through specific logbooks. In general, m-health apps are often used to facilitate communication between patients and health care providers. It is relevant that according to recent surveys the majority (81%) of m-health app publishers are now integrating tools or devices in at least some of their portfolios. In addition, 50% of developers are offering tools (Application Programming Interface or API) to facilitate incorporation of third-party health and fitness data points and 49% connect their apps to EHR or EHR functionalities, allowing data exchange and easy integration between different apps and services (49).

### Benefits and Drawbacks of m-health

m-health shares many of the advantages of telehealth. However, given the fact that smartphones, at variance from personal computers, are always on and connected, and that they are carried along by patients as a part of modern lifestyle, make them an ideal mean to deliver connected health.

Specifically, smartphone apps can empower patients and promote self-management, encouraging greater patients' participation in medical decision making. In general, m-health enhances communication between patient and physician or care team with the potential for improvement of patient's adherence to treatment and physician's inertia. It can be a winning solution because it allows remote monitoring of patients difficult to reach or needing strict surveillance, by making use of a relatively cheap, easy to afford and convenient technology, which relies on existing mobile networks. m-health has also the potential to improve control of risk factors and health status, particularly for patients with chronic conditions such as arterial hypertension, diabetes, or heart failure.

Unfortunately, m-health possesses some drawbacks which limit its diffusion. As a matter of fact, at present, only 25% of doctors recommend the use of m-health to monitor patients and 42% of doctors worry that m-health apps will make patients too independent. Some of the challenges of m-health are related to the mobile technology *per se*: although coverage of the popular 3G technology increased from 75 to 87% in recent years, network coverage and quality of the connectivity still remains limited in rural and remote areas (45). Affordability of Internet-enabled mobile technologies remains one of the biggest barriers for consumers in some areas of the globe. There is still a significant proportion of people who are unconnected because they lack the skills to access and engage with mobile technology, particularly in low- and middle-income countries, and among women (gender gap). The distribution and access of content and services are often limited by language barriers because some applications and some contents are delivered only in local languages. Specifically concerning m-health applications, there is currently no proper regulation, standardization, and validation of the development process of the technologies. Only the US Food and Drug Administration has issued specific guidelines for developers, but yet challenges remain (50, 51). There are no standardized methods for the quality evaluation of m-health apps and the information provided by these tools may be inaccurate. Most of the m-health apps are not yet considered medical devices (most are enlisted in the “fitness” or “wellness” category of the app stores). For instance, only a few of the m-health apps for hypertension, which are used to measure BP or to provide advise on high BP management, can be regarded as accurate and safe for clinical use (as further discussed). Finally, m-health presents potential privacy and security issues related to the exchange of sensitive data. Not all m-health apps are compliant with privacy regulations and thus there is no guarantee that a user's health information will be protected or that users will be notified in case of a data breach.

### m-health Apps for Hypertension Management

**Figure 4:**
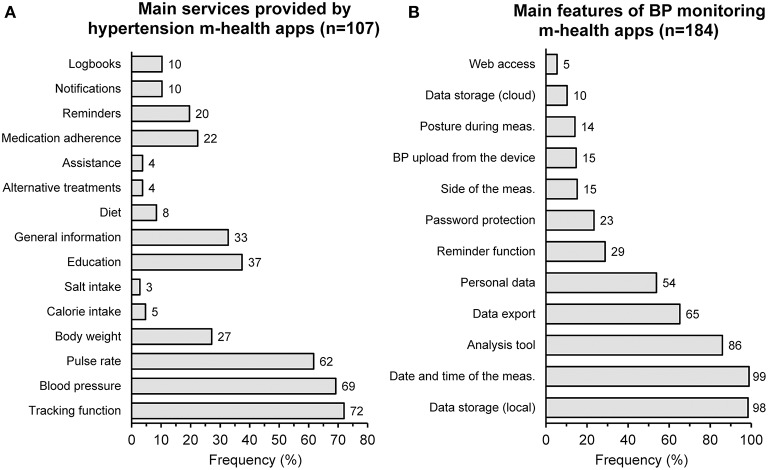
Main services provided by hypertension m-health apps according to a survey of 107 different products **(A)** and main characteristics of m-health apps used for blood pressure (BP) monitoring according to another survey of 184 applications **(B)** [redrawn from (52) and (53) by permission].

A review of mobile health applications designed for the management of hypertension has revealed that the most popular downloaded and used apps are those with tracking facilities (Figure 4A) (52). They help patients to monitor various physiological parameters, such as BP and heart rate, and change their lifestyle through diet and exercise, as well as remind patients to take their medications. Of the 77 tracking apps evaluated by Kumar et al. (72% of the total reviewed apps), 69% were capable of detecting BP and heart rate, 27% measured weight and/or body mass, 5% daily calories and 3% salt intake. None of the 57 apps made for the iOS operating system was devised as a BP-measuring tool, whereas 7 of the 50 (14%) apps made for the Android market could work as a BP monitor. Android apps with BP or heart rate measuring capabilities were downloaded 2.4 million times by consumers and received high ratings. Unfortunately, the quality of the development process for these apps was poor: only 3% of the apps were developed by healthcare professional agencies, no BP-measuring app was validated against a gold standard, nor obtained approval for use as a medical device.

Despite the huge variety of apps for hypertension management and the increasing use of smartphones and tablets, according to a recent US survey, m-health apps seem to be used less among people with self-reported history of hypertension (54). According to the data collected by Langford et al. in 3,285 respondents, of which 1,460 reporting a history of hypertension, tablet or smartphone ownership was significantly (*p* <0.001) lower in participants with (55% and 66%) than in those without hypertension (86% and 68%). Compared to those without a history of hypertension, participants with a history of hypertension were less likely to have health-related apps on their smartphones (30% vs. 45%, *p* < 0.001) and less often reported that mobile devices helped them achieve a health-related goal (63% vs. 72%, *p* = 0.01).

### Smartphone-Based Devices and Applications for BP Monitoring

Smartphone apps used to measure BP can be divided into three major categories, as summarized in Table 5.

**Table 5:** Main categories of m-health apps for BP measurement management.

**Category**	**Advantages**	**Disadvantages**
Manual insertion of values by the user	Flexibility Widely available in digital stores Not tied to a specific device No need to perform the recording simultaneously to the measurement	High risk of errors in the transcription of values Omission of data reporting
Automated data transmission through wireless pairing with an oscillometric BP measuring device	Widely available from the manufacturers Validated devices with high accuracy No particular technical skill required Low risk of errors and data loss due to automatic transmission High portability	Tied to a specific brand or model Inability to pair with the BP monitor and upload data
Apps or add-ons that turn the smartphone into a measuring device (usually “*cuffless*”)	No need for a device other than the smartphone Always available No need of a cuff Relatively easy to use	Lack of standardization Poor accuracy and reliability Lack of validation Possible inaccuracy due to incorrect positioning of the sensor

*BP, Blood Pressure*.

m-health apps that allow to record and store BP values manually entered by users are the simplest and most popular ones. The main advantages of these apps are wide availability and flexibility, as they do not require the measurements to be performed at the time of the data entry and they are not tied to a single device type. Errors in the transcription of BP values or omission of data reporting are the obvious drawbacks of these popular apps.

A second category includes those apps which can allow BP data download from a wireless measuring device paired to the phone. These apps are usually developed and made available in the app store by the device manufacturers. For this reason, they are usually highly reliable and linked to accurate and validated devices. They also bring a very low risk of errors or data loss during automatic transmission and they possess portability for multiple platforms (commonly the same app is developed at least for Android and iOS). Unfortunately, these apps can only be used with specific brands or models of BP measuring devices and are not interchangeable among different models or brands. Sometimes, pairing between the app and the device may fail, particularly when multiple wireless devices are linked to the same smartphone.

Finally, a very popular but still highly unreliable category of m-health apps used for measuring BP are those ensuring a “*cuffless*” measurement, namely those that are able to measure BP without the need of an external device equipped with an arm or wrist cuff. These tools usually rely on the native functions of the smartphone, with or without add-ons. The most interesting tools are those making use of a photoplethysmogram sensor-based cuffless BP technology, which is based on optic measurements of the pulse wave from the finger, made with sensors added to the smartphone's case or with the smartphone's camera or both. In case of measurements made with the smartphone camera, the patients merely press the finger against the optics and he/she receives the measurement. Cuffless devices appear to be the most promising among m-health based BP monitoring devices because they are particularly easy-to-use, they can be used everywhere without the need of a cuff or a dedicated device, an aspect which is important for patients with busy schedules or who are frequently traveling and cannot carry around a conventional blood pressure measuring machinery.

Unfortunately, these devices are also prone to frequent artifacts and high inaccuracy if not properly used, and very little research has been performed comparing the accuracy of BP readings obtained from smartphone-based cuffless technology with measurements obtained with a reference gold standard (auscultatory upper-arm measurement) or with an oscillometric automatic upper arm device. The few validation studies performed so far with cuffless (photoplethysmographic) measuring devices suggest that this technology may have a promising future, but underscore that the few commercially available smartphone apps or add-ons which have been tested have overwhelming failed validation trials for accuracy in BP measurement, whereas heart rate estimation is acceptable, at least in the adult (55–61) (Table 6).

**Table 6:** Validation studies assessing the accuracy of BP measurements provided by cuffless devices or apps for smartphones.

**Device features [references]**	**Test technology**	**Reference technology**	**Number of subjects**	**Type of subjects**	**Mean test—reference difference ± SD (mmHg)**		**Results**
					**SBP**	**DBP**	
Add-on for Android smartphone (56)	Oscillometry applied to finger photoplethysmography (device to be affixed to the back of the smartphone)	Oscillometric upper-arm device	35	Healthy subjects	+3.3 ± 8.8	−5.6 ± 7.7	Large bias and precision error between cuffless smartphone BP and upper-arm oscillometric BP recording The device did not output any BP measurement for 3 of the 35 subjects studied About 60% of the measurements were successful and the device yielded multiple BP measurements for about 80% of the users
Add-on for iPhone (57)	Oscillometry applied to finger photoplethysmography (device to be affixed to the back of the smartphone)	Oscillometric upper-arm device	20	Healthy subjects	−4.0 ± 11.4	−9.4 ± 9.7	Large bias and precision error between cuffless smartphone BP and upper-arm oscillometric BP recording The device did not output any BP measurement for 2 of the 25 subjects studied
Add-on for smartphone (58)	Finger photoplethysmography (sensor external to the smartphone)	Oscillometric upper-arm device and auscultatory method	172	Healthy subjects	+0.3 ± 6.6	−1.0 ± 5.4	Relatively good accuracy of the cuffless device at rest In dynamic conditions (during BP rise or lowering) the accuracy worsened >70% of the 35 subjects using the device during night sleep felt it less uncomfortable than an ordinary upper-arm cuff device Good short-term reproducibility of the device (ICC 0.950 for SBP and 0.903 for DBP)
Preventicus BP smartphone algorithm embedded in an app for iPhone (59)	Finger photoplethysmography through smartphone's camera	Oscillometric upper-arm device	32	Pregnant women	+5.0 ± 14.5	N.A.	The device did not fulfill the requirements of the 2010 ESH IP The algorithm overestimated SBP in low range and underestimated it in the medium range
Instant Blood Pressure app for iPhone (60)	Finger photoplethysmography through smartphone's camera	Oscillometric upper-arm device	85	Healthy (40) or hypertensive subjects (45)	+12.4 ± 10.5	+10.1 ± 8.1	The app underestimated higher BP and overestimated lower BP and was found inaccurate according to BHS protocol It had a lower sensitivity for high BP readings and inappropriately classified 78% of users with hypertension as normotensive
Instant Blood Pressure app for iPhone (61)	Finger photoplethysmography through smartphone's camera	Oscillometric upper-arm device	100	Healthy subjects	−0.6 ± 12.8	+7.2 ± 9.2	The SBP values from the application were not significantly different from those from the reference monitor, but had wide limits of agreement, not recommending clinical utilization DBP values were inaccurate ICC 0.688 for SBP and 0.377 for DBP
Instant Blood Pressure Pro app for iPhone (61)	Finger photoplethysmography through smartphone's camera	Oscillometric upper-arm device	100	Healthy subjects	+0.3 ± 15.3	+7.4 ± 11.3	The SBP values from the application were not significantly different from those from the reference monitor, but had wide limits of agreement, not recommending clinical utilization DBP values were inaccurate ICC 0.401 for SBP and 0.257 for DBP

*SD, Standard Deviation; ICC, Intraclass Correlation Coefficient; SBP, Systolic Blood Pressure; DBP, Diastolic Blood Pressure; BP, Blood Pressure; ESH IP, European Society of Hypertension International Protocol; BHS, British Hypertension Society*.

A content analysis of apps specifically designed for BP monitoring has been recently published by Jamaladin et al. (53). The authors scrutinized 184 apps, of which 104 Android and 80 iOS apps. A summary of the main characteristics of the included apps is reported in Figure 4B. The overall quality score, on a scale from 1 to 5, was 2.63 for Android and 2.64 for iOS. The app features with the most positive influence on the quality score were the ability of using the cloud for data storage, wireless data upload from BP monitors, ability to export data, ability to analyze data, ability so set and send reminders, and ability to record personal data together with BP, such as age and body weight. Unfortunately, the information or education feature was often absent or of poor quality in the apps evaluated. Only 2 apps were developed by a university or non-governmental organizations, seven stated the involvement of medical experts in the development process and none of the apps was evaluated with results published in the literature.

### Evidence of Efficacy of M-health for Improving BP Control and Adherence

A consistent number of studies have evaluated the impact of m-health interventions in the management of hypertension and found the m-health interventions effective (62). However, in most of these studies, the intervention was based on text messaging and very few used mobile phone applications and wireless devices. Thus, although the number of smartphone apps offered to patients with hypertension is growing rapidly, the evidence supporting the effectiveness of different m-health strategies is scarce. Long-term cardiovascular outcomes data and analyses relating to cost-effectiveness are also lacking.

Two recent systematic reviews evaluated the potential effect of mobile apps for hypertension management. One paper published by Alessa et al. included 21 studies with 3,112 participants and assessed the effectiveness of 16 different apps in lowering BP, as well as their usability and patients' satisfaction with their use (63). Ten of the 14 studies (of which 6 randomized controlled and 4 non-randomized studies) that assessed the effectiveness of the apps in lowering BP, reported that using the apps led to significant decreases in BP and thus seemed to be effective in the self-management of hypertension. Only 5 (2 randomized controlled and 3 non-randomized studies) of these 10 studies had a low–moderate risk of bias, namely an acceptable quality. The use of the app was generally highly accepted by participants in all the nine studies that assessed this measure. The satisfaction ranged from 7.2 to 9.8 for studies using a 10-point satisfaction rating scale, and from 3.1 to 4.8 for studies utilizing a 5-point satisfaction rating scale. Two studies explored the usability of the apps and the participants reported that the apps were easy to use, convenient, helpful in effectively communicating with healthcare providers and in hypertension management, including medication adherence and adjustment, and helped increase their active role in care, health awareness, and motivation.

In another systematic review, Mohammadi et al. evaluated 13 studies with an app-based intervention lasting <1 year on hypertensive or high-risk individuals. The review confirmed that m-health apps are capable of improving an individual's BP and its management (in particular adherence to medication) (64). m-health solutions also hold enormous potential to support hypertensive disorders during pregnancy, as suggested by Rivera-Romero et al. in their meta-analysis of 11 studies (65). Very positive results in the improvement of maternal health and acceptability of solutions were found, although most of the studies included in the review involved a small number of participants, only 4 used sensors for measuring physiological parameters, only two specifically used BP sensors and none were complete clinical studies.

The improvement in medication adherence among people with hypertension, following an m-health app intervention, seems to be the most important effect of m-health. Xiong et al. examined 21 studies based on m-health interventions including high intervention intensity, multifactorial components, and patient-centered approaches with tailored content and interaction (66). All studies documented a trend to an improvement in medication adherence, with 12 studies reporting a superiority of the intervention over the usual care. Twelve studies also found a benefit in terms of BP control for interventions based on m-health.

Thus, current evidence of the efficacy of m-health apps in improving BP and compliance to treatment is encouraging, but most studies had a small size, a short duration, and not all results were statistically significant. Additionally, most of the studies appear to be inconclusive regarding which combinations of functionalities would be most effective, because of variation in the quality of the studies, though current data suggest that apps incorporating more comprehensive functionalities are likely to be more effective. Future research should attempt at investigating the most effective functionality and assessing the sustainability and generalizability of m-health interventions.

## Benefits and Challenges of Effective Implementation of E-health in Hypertension Management

The application of e-health and telehealth to hypertensive patient care brings several important benefits (18, 19).

Health information technologies may help to build and maintain an enduring and long-term relationship between patients and their healthcare providers. e-health and telehealth may help empower hypertensive patients, and promote self-management, with improvement in patient's medical condition. Digital interventions can help individualize the physician-patient relationship, and thus improve BP and cardiovascular risk control. Remote patient monitoring through telemedicine allows physicians and health facilities to reach distant patients and increase the number of served patients, with consistent time savings, yet maintaining high-quality standards of delivered care. In particular, telemedicine improves the tracking and communication of various biometric information, actively engaging the patients in their care. In the case of hypertensive patients, telemedicine services can be used to easily and rapidly communicate to the referring doctor the occurrence of acute symptoms or sudden BP raises. Finally, telehealth services grant hypertensive patients the access to diagnostic procedures (e.g., ambulatory blood pressure monitoring or electrocardiogram facilities in community pharmacies linked to telemedical reporting services) that might not be available otherwise, without the need to cover long distances.

The integration of e-health and telehealth into the health care system is not without challenges. The lack of adequate infrastructures, the relatively high costs of the services, the lack of reimbursement, and privacy and legal issues are major barriers to the adoption of connected health solutions in the daily practice of healthcare professionals and patients. The development of telehealth programs requires investment in infrastructure, including the purchase and maintenance of computer hardware and related software, as well as secure means for data transmission, compliant with current privacy regulations. Less complex, user-friendly and cheap tools, possibly integrated into mobile phones, tablets, or home appliances, should be preferred. Indeed, an effective information technology system, with interoperability with existing systems, and supporting infrastructure, may facilitate diffusion and penetration of telemedicine. Additional costs are those for recruitment and licensing of appropriate professionals for service management and those of providing training and support services to staff and users. Lack of reimbursement of services and consultations plays a critical role in preventing or delaying the development and diffusion of e-health solutions.

Privacy and security issues are important when data are traveling through the web. Since telemedicine involves collection and sharing of patients' health information, safeguards are needed to be put in place to secure the privacy and safety of these data, in order to minimize the risk of data breach. Compliance with privacy regulations is mandatory in order to guarantee users about the integrity of their health data.

The educational level and features of the patients targeted for the intervention is also important as well as the promotion of the clinical usefulness of e-health services among doctors and patients. Behavioral and organizational barriers are also major concerns to the diffusion of e-health and telehealth programs. Resistance to adopt new models of care affects both patients and healthcare workforces. In particular, physicians are important gatekeepers in telemedicine adoption and diffusion and their endorsement is an important pre-requisite for the success of telemedicine programs. In order to facilitate diffusion of telehealth services, efforts should be dedicated to their integration into existing organizational structures and to provide institutional support to execute these services.

## Perspectives

As healthcare is moving from a traditional in-office disease-centered to any-time, any-place, continuous and personalized care, digital health and connected care gain momentum.

It is expected that telehealth will boom in the next years because of the high demand by patients and doctors; providing clinical care at a distance, increasing accessibility and eradicating potential delays will improve the quality of care and boost patient satisfaction and overall engagement.

Artificial intelligence will be one of the most important keys to digital health transformation. Networks based on tools with machine learning capabilities will pave the way to data-driven decision support services. These tools will assist physicians to make accurate diagnoses, predicting potential therapeutic candidates for specific patient care and mining medical data stored in EHRs to improve healthcare service delivery. Artificial intelligence applications, such as predictive analytics for patient monitoring will also provide significant financial savings.

Robotic care will help doctors to actively manage their patients at a distant location, administering drugs, examining patients, or performing surgical operations while being seated at his/her computer. Drones will also help to transport pharmacy supplies or lab samples and to move patients around.

Hand-held portable diagnostic devices or complex health wearables with mobile connectivity (so-called “*medical tricorders*”) will help to diagnose multiple medical conditions with a consumer-friendly interface. In particular, devices that monitor blood glucose, BP, heart rate, physical activity, and sleep will gain popularity among patients and healthcare providers.

Web-based augmented and virtual reality applications will be used for medical education, to train healthcare professionals, or during a diagnostic examination or surgery to determine the exact location of organs and vessels. This technology is also currently used by patients to manage some mental disorders, but there is potential for expansion toward more advanced applications in another field, including hypertension management (e.g., education about BP measurement procedures).

Remote 3D printing may allow to print tissues (e.g., skin) and, in the future, even organs. 3D-printed medicines may allow alteration of daily dosage and enable personalized treatments by customizing formulations of the drugs.

Finally, the introduction of blockchain in remote health management will help to provide transparency and to eliminate third-party intermediaries, ensuring the delivery of value-based healthcare at low costs.

## Conclusions

Connected health is progressively gaining a key role in the management of hypertensive patients, having the potential to improve the quality of the delivered care, to increase adherence to antihypertensive treatment and to more effectively control BP. When properly implemented, the broad adoption of telehealth and m-health solutions in the field of hypertension management has the potential to extend screening across populations of patients and help achieve the important policy goals of improving access to high quality and efficient health care to as many as possible persons at risk.

## Author Contributions

SO devised the original idea of the work, made the search of the literature and wrote the manuscript. The author meets the ICMJE criteria for authorship for this manuscript, takes responsibility for the integrity of the work as a whole, and has given final approval to the version to be published.

### Conflict of Interest Statement

SO is scientific consultant of Biotechmed Ltd., provider of telemedicine services.
